# Molecular diagnosis of primary liver cancer by microsatellite DNA analysis in the serum

**DOI:** 10.1038/sj.bjc.6600649

**Published:** 2002-12-02

**Authors:** Y-C Chang, C-L Ho, Helen H-W Chen, T-T Chang, W-W Lai, Y-C Dai, W-Y Lee, N-H Chow

**Affiliations:** Department of Surgery, College of Medicine, National Cheng Kung University Hospital, 138 Sheng-Li Road, Tainan 704, Taiwan, Republic of China; Department of Pathology, College of Medicine, National Cheng Kung University Hospital, 138 Sheng-Li Road, Tainan 704, Taiwan, Republic of China; Department of Radiation Oncology, College of Medicine, National Cheng Kung University Hospital, 138 Sheng-Li Road, Tainan 704, Taiwan, Republic of China; Department of Internal Medicine, College of Medicine, National Cheng Kung University Hospital, 138 Sheng-Li Road, Tainan 704, Taiwan, Republic of China; Institute of Molecular Medicine, College of Medicine, National Cheng Kung University Hospital, 138 Sheng-Li Road, Tainan 704, Taiwan, Republic of China

**Keywords:** hepatocellular carcinoma, cholangiocarcinoma, microsatellite, loss of heterozygosity, molecular diagnosis

## Abstract

Frequent loss of heterozygosity of microsatellites markers on specific chromosomal region have been reported in various types of primary human cancer. The same loss of heterozygosity has also been identified in the matched plasma/serum DNA. Using 109 microsatellite markers representing 24 chromosomal arms, we have examined the loss of heterozygosity in 21 cases of hepatocellular carcinoma, six of cholangiocarcinoma, and 27 cases of chronic hepatitis or cirrhosis. All cases of the hepatocellular carcinoma showed deletion from two to 10 chromosomal arms, while deletion of chromosomes from two to eight regions was detected in five of six cholangiocarcinoma patients. One or more loss of heterozygosity in the paired serum DNA could be detected in 16 of 25 (76.2%) hepatocellular carcinoma patients. In contrast, no alterations in serum DNA test could be found in cholangiocarcinoma patients. Five of seven (71.4%) hepatocellular carcinoma patients with alpha-fetoprotein levels less than 20 ng ml^−1^ produced positive serum DNA test. The profiles of 19 microsatellite markers gave a 100% positive predictive value and an 80.8% negative predictive value for hepatocellular carcinoma. In conclusion, we have determined a profile of microsatellite markers appropriate for differential diagnosis of primary liver cancer. The discovery may permit a high-throughput screening of hepatocellular carcinoma at an early stage of disease.

*British Journal of Cancer* (2002) **87**, 1449–1453. doi:10.1038/sj.bjc.6600649 www.bjcancer.com

© 2002 Cancer Research UK

## 

Primary liver cancer ranks fifth in frequency among all malignancies in the world with an estimated number of 437 000 new cases in 1990. The vast majority of primary liver cancer is hepatocellular carcinoma (HCC), a malignant tumour derived from hepatocytes. HCC is increasing in many countries, particularly in areas where hepatitis C virus infection is more common than hepatitis B virus infection (El-Serag and Mason, 1999). The cholangiocarcinoma (CC), an intrahepatic malignant tumour which arises in the epithelium of the bile ducts, is the second common hepatic tumour. Its occurrence is largely high in areas where liver fluke infestation or intrahepatic stones is endemic, such as Hong Kong, Thailand and Taiwan (Okuda *et al*, 1977).

The prognosis of HCC or CC is usually poor, with 3-year survival rates estimated at 44.9% and 34% after hepatic resection, respectively (Okuda *et al*, 1977). In patients with symptomatic HCC, the tumours are usually either too large and/or with local invasion or distant metastasis. Therefore, only 10–20% of patients could be treated with potentially curative therapy, while the remaining patients can only receive non-surgical therapy, such as a transcatheter arterial embolisation or other local ablation treatments with a less satisfactory outcome. The patients with CC often present with obstructive jaundice at the stage of advanced stage. Currently, resection offers the only chance of cure and the best chance of long-term survival.

In order to improve the chances of successful treatment and patient survival, great effort has been devoted to detect HCC in the early stage when it is ‘small’, for example smaller than 3 cm, and where liver function reserve is good enough for curative therapy. Measurement of serum α-fetoprotein (AFP) and hepatic ultrasonography are widely used as surveillance modalities for high-risk patients. Elevated levels of AFP, with normal range set at 20 ng ml^−1^, are detected in up to 75% of HCC patients, and values above 400 ng ml^−1^ are considered diagnostic for HCC (Ebara *et al*, 1986). However, 35% of HCC smaller than 3 cm may secrete little or no AFP into the circulation and thus will not be identified by this test (Ebara *et al*, 1986). As a result, measurement of AFP alone is not an ideal test for early diagnosis of HCC.

Recent advances in tumour genetics reveal that the genesis and progression of tumours follow an accumulation of multiple genetic alterations, including inactivation of tumour suppressor genes and/or activation of proto-oncogenes. Frequent loss of heterozygosity (LOH) of microsatellites markers on specific chromosomal region have been reported in various types of human cancer. In addition, nucleic acid-based molecular techniques have successfully identified the same LOH in the paired plasma/serum DNA from patients with diverse types of epithelial cancer, such as lung (Chen *et al*, 1996; Sozzi *et al*, 1999, 2001), head and neck (Coulet *et al*, 2000; Nunes *et al*, 2001), colon (Hibi *et al*, 1998), breast (Chen *et al*, 1999; Silva *et al*, 1999), kidney (Goessl *et al*, 1998; Eisenberger *et al*, 1999). The results indicate that microsatellite DNA analysis may have potential as a non-invasive test for cancer diagnosis. In this pilot study, we have searched for the genetic alterations, using a total of 109 microsatellite markers representing 24 chromosomal regions, in the serum DNA of patients with primary liver cancer, i.e. HCC (*n*=21) and CC (*n*=6). The results suggest that microsatellite serum DNA analysis may be a valuable, non-invasive method for the early detection of primary liver cancer.

## MATERIALS AND METHODS

### Sample collection and DNA preparation

Primary tumour from 21 cases of HCC and six cases of CC were collected from the Department of Surgery, National Cheng Kung University Hospital, Tainan, Taiwan between September 1998 and March 2000 (Tables 1

**Table 1:**
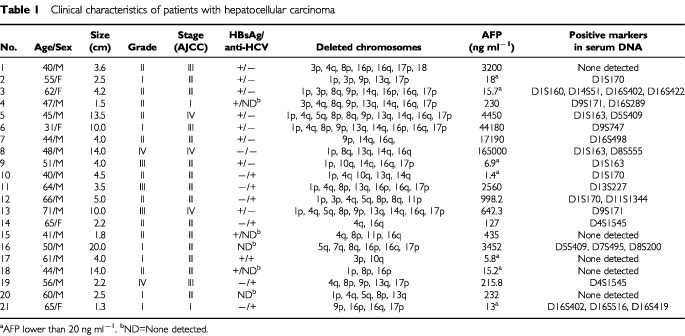
Clinical characteristics of patients with hepatocellular carcinoma

and 2

**Table 2:**
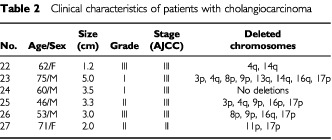
Clinical characteristics of patients with cholangiocarcinoma

). The tumour tissue was obtained immediately after surgical resection and stored at −80°C, and then microdissected as described in detail previously (Eisenberger *et al*, 1999). We also enrolled 27 patients (age- and sex-matched) with a biopsy diagnosis of chronic hepatitis (*n*=20) or cirrhosis (*n*=7) as controls. Both peripheral blood lymphocytes and serum samples were obtained with written informed consent. Briefly, DNA was extracted from peripheral blood lymphocytes, tumour and matched serum samples by digestion with 1% SDS/proteinase K (BD Biosciences Clontech, CA, USA) overnight followed by phenol-chloroform extraction and ethanol precipitation. Each tumour was reviewed for histologic grading according to the Edmondson classification (1958). Clinical staging was determined according to the tumour-node-metastasis staging protocol of the American Joint Committee on Cancer (1997) with surveys of the clinical details, image studies, and pathologic data. The clinical characteristics, including AFP, hepatitis B surface antigen (HBsAg) and hepatitis C virus antibody (anti-HCV), were obtained from medical records.

### Analysis for allelic loss

Microsatellite markers from selected chromosomal arms for PCR analysis were obtained from Research Genetics (Huntsville, AL, USA). The marker pairs selected for HCC (74 markers from 17 chromosomal arms) included 1p (D1S160, D1S163, D1S170, D1S186, MYCL), 3p (D3S1317), 4p (D4S394), 4q (D4S398, D4S395, D4S392, D4S422, FGA, FABP2, D4S427, D4S415, D4S1615, D4S1554, D4S1426, D4S1598, D4S620, D4S1566, D4S1545, D4S2920, D4S2943, D4S2954), 5q (D5S409), 6q (D6S264), 8p (D8S261, D8S277), 8q (D8S85, D8S200, D8S555, D8S283), 9p (D9S169, D9S1747, D9S104), 10q (D10S109), 11p (D11S554, D11S436, D11S1344, D11S932, D11S1324), 11q (D11S938, D11S29), 12p (D12S93), 13q (D13S171, D13S153, Rb1, D13S133, D13S227, D13S159, D13S166, D13S168), 14q (D14S72, D14S51), 16p (D16S419, D16S409, D16S3106, D16S498), 16q (D16S415, D16S408, D16S512, D16S289, D16S402, D16S516, D16S422, D16S413), and 17p (D17S520, D17S1176, TP53, D17S513, D17S578, D17S796, D17S849) based on previous reports (Yeh *et al*, 1996; Nagai *et al*, 1997; Piao *et al*, 1998; Fujii *et al*, 2000; Okabe *et al*, 2000; Yeh *et al*, 2001). The marker pairs selected for CC (35 markers from 16 chromosomal arms) included 2p (BAT-26), 3p (D3S3667, D3S1578, D3S3582, D3S3560, D3S1581, D3S3729, D3S1588, D3S3648), 4 (D4S415, D4S413), 5q (D5S323, D5S417), 6p (D6S263), 6q (D6S292), 7q (D7S495, D7S486), 9p (D9S747, D9S171), 11p (D11S907, D11S569), 14q (D14S1436), 16q (D16S3094, D16S511, D16S534, D16S520), 17 (D17S695), 18q (D18S67, D18S51, D18S535), 20 (D20S85), 21q (D21S1245, D21S1436, D21S1270), and Xp (DXS538) (Ding *et al*, 1993; Fujii *et al*, 2000). All of the tumours, irrespective of histopathology, were analysed for a total of 109 microsatellite markers (74 markers for HCC and 35 markers for CC) to confirm the specificity of chromosomal alterations as well as their presence in the serum as tumour markers.

**Figure 1:**
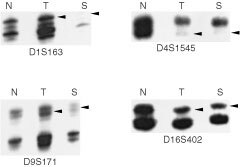
LOH was scored for if the tumour allele (T) had a greater than 30% reduction in intensity compared to the corresponding allele in the normal control (N). Identical loss, highlighted by arrowhead, was also demonstrated in the matched serum (S).

Prior to amplification, one primer from each pair was end-labelled with [γ-^32^P]ATP (20 μCi; Amersham Pharmacia Biotech Inc., NJ, USA) and T4 kinase (New England Biolabs Inc., MA, USA) (Eisenberger *et al*, 1999). The amplification conditions for polymerase chain reaction (PCR) were 1× buffer (GibcoBRL, NY, USA), 1.5 mM MgCl_2_, 0.6 mM dNTP, 5 pmol of each primer, 1.0 μl γ-^32^P-labelled primer, 4 μl DNA, and 0.1 μl of Taq DNA polymerase (GibcoBRL, NY, USA) in a total of 10 μl. PCR for each primer set was performed for 35 cycles consisting of denaturation at 95°C for 30 s, annealing at 50–60°C for 1 min, and extension at 70°C for 1 min (Perkin-Elmer Gene Amp PCR System 9600, Perkin-Elmer Corporation, MA, USA). The reaction products were separated on 7% urea- formamide-polyacrylamide gels (GibcoBRL, NY, USA) and exposed for 12–24 h. For informative tumours, LOH was scored if the tumour allele demonstrated a greater than 30% reduction in intensity compared to the corresponding normal control visually as previously described (Figure 1

) (Eisenberger *et al*, 1999). Each LOH was confirmed by two independent experiments. Allelic loss was determined by observation of LOH at any informative marker mapped to the same chromosomal region. Then, LOH analysis was performed on matched serum samples.

### Statistical analysis

Allelic status was correlated with clinical and pathologic factors of HCC, such as histological grading, disease stage, tumour size, serum AFP levels, portal vein thrombosis and satellite nodule formation by Fisher's exact test. To evaluate the diagnostic accuracy in the microsatellite analysis, we considered four events: (A) positive test: presence of LOH in the primary tumour and in the matched serum DNA (true positive); (B) positive test: absence of LOH in tumour but presence of LOH in patient serum (false positive); (C) negative test: presence of LOH in tumour but absence in paired serum samples (false negative); and (D) negative test: absence of LOH either in tumour or in paired serum samples (true negative). Decision diagnostic criteria were obtained from the results A, B, C, and D. Test sensitivity is equal to A/A+C. Test specificity is equal to D/D+B. Test accuracy is equal to: A+D/A+B+C+D. Predictive positive result or positivity prediction is equal to A/A+B. Predictive negative result or negativity prediction is equal to D/D+C.

## RESULTS

All of the HCC cases showed deletion from two to 10 chromosomal arms (Table 1). The highest incidence was 16q (66.7%), followed by 1p (57.1%), 4q (57.1%), 8p (52.4%), 17p (52.4%), 13q (47.6%), 14q (42.9%), 9p (33.3%), 16p (33.3%), 3p (28.6%), 8q (28.6%), 5q (23.8%), 10q (14.3), 11p (9.5%), 18 (4.8%), and 7q (4.8%), respectively. The allelic losses were compared with biologic indicators of HCC, such as histological grading, tumour stage, size, and AFP serum levels (Table 1). A positive correlation was observed for 4q with AFP greater than 20 ng ml^−1^ (*P*=0.02), and 13q with satellite lesion (*P*=0.04) (data not shown). There was a trend toward positive association of 8p or 13q loss with advanced tumour staging (*P*=0.06), and 8p loss with AFP greater than 20 ng ml^−1^ (*P*=0.06). LOH on 14q and 16q occurred more frequently in hepatitis C virus-negative patients (*P*<0.05, respectively). Otherwise, patterns of allelic loss did not relate to histological grading or tumour size (*P*>0.1, respectively). In addition, novel alleles indicative of microsatellite instability were found in three patients (cases 2, 3 and 7, respectively) at chromosome 2, 13 and 16, respectively (data not shown).

As for tumour diagnosis, a total of 16 of 21 (76.2%) HCC patients were found to have LOH in serum DNA from one to four microsatellite markers (Table 1). The presence of LOH in the serum DNA did not relate to histological grading, staging or tumour size (*P*>0.1, respectively). If the cut-off values were set at AFP 400 ng ml^−1^, negative serum LOH test was observed in two of 10 (20%) patients with abnormal AFP level (cases 1 and 15), and in three of 11 (27.3%) patients having AFP level within normal limits (case 17, 18 and 20). In contrast, five of seven (71.4%) patients (cases 2, 3, 9, 10 and 21) having AFP less than 20 ng ml^−1^ had positive LOH test. The results imply that microsatellite DNA analysis may have potential as a non-invasive diagnostic test for the early detection of HCC, especially for those who having AFP below reference range. However, none of three cases showing microsatellite instability in primary tumours had the same alterations detected in the serum DNA (data not shown).

With regard to cholangiocarcinoma, five of six patients belong to advanced stage of tumour (Table 2). Deletion of chromosome was detected from two to eight regions in five of six patients. But, no any alterations in serum DNA test could be found, despite of the fact that some common allelic losses were observed between HCC and CC. The results seem to imply a limited potential of the microsatellite analysis in diagnosing of CC.

Altogether, the data of this pilot study indicate that microsatellite analysis of serum DNA could successfully detect around three-quarters (76.2%) of HCC by screening the following profile of microsatellite markers, i.e. D1S160, D1S163, D1S170, D4S1545, D5S409, D7S495, D8S200, D8S555, D9S171, D9S747, D11S1344, D13S227, D14S51, D16S289, D16S402, D16S419, D16S422, D16S498, D16S516. The positive predictive value was 100%, and negative predictive value was 80.8%.

## DISCUSSION

Currently, only ultrasound-guided needle biopsy yields diagnostic material and allows a definitive differential diagnosis between HCC and CC (Tsuji *et al*, 1999). Measurement of serum AFP and hepatic ultrasonography are widely used as surveillance modalities for patients with high-risk of liver cancer. In patients with HCC, however, around one-third of small tumour (<3 cm) was shown to secrete little or no AFP into the circulatory system (Ebara *et al*, 1986). As a result, it is mandatory to explore supplementary tumour marker for early detection of HCC.

In this study, we detected one or more LOH in the serum DNA in three-quarters of HCC patients. The results are comparable to some prior reports showing microsatellite alterations in the plasma/serum from cancer patients (Chen *et al*, 1996, 1999; Goessl *et al*, 1998; Hibi *et al*, 1998; Eisenberger *et al*, 1999; Silva *et al*, 1999; Sozzi *et al*, 1999, 2001; Coulet *et al*, 2000; Nunes *et al*, 2001). However, the sensitivity of microsatellite DNA test greatly depends on the kind of cancer studied. For example, the detection rate was 71% for small cell carcinoma of the lung (Chen *et al*, 1996), from 60 to 63% for renal cell carcinoma (Goessl *et al*, 1998; Eisenberger *et al*, 1999), between 48 and 66% for breast cancer (Chen *et al*, 1999; Silva *et al*, 1999), from 40 to 45% for non-small cell lung cancer (Sozzi *et al*, 1999, 2001), between less than 2 and 18.7% for head and neck cancer (Coulet *et al*, 2000; Nunes *et al*, 2001) using a set of primer pairs.

In contrast, the lack of tumour-specific LOH in the serum of CC patients appears to correspond to the observation reported for colon cancer (0%) (Hibi *et al*, 1998), though these patients may still have very low levels of tumour-derived DNA. Nonetheless, the difference appears to support the propensity of HCC to spread to vascular system (portal vein and hepatic vein), whereas perineural invasion and lymphatic involvement are frequently present in CC at an early stage (Sasaki *et al*, 2001). Taken together, the data support the detection of circulating tumour-derived DNA as a supplementary tumour marker in the differential diagnosis of primary liver cancer.

Of particular interest, LOH in the serum DNA could be detected in five of seven HCC patients with serum AFP less than 20 ng ml^−1^, the normal range for the healthy adults. Previous study found that approximately one third of small HCC (less than 3 cm) may secrete little or no AFP into the circulation (Ebara *et al*, 1986). Our data imply that the limitation of low specificity of AFP in diagnosing small HCC might be compensated by microsatellite analysis on cell-free DNA in cancer patients. It will be especially valuable in patients with poor liver function or ascites, in which further diagnostic investigation is prohibited. A prospective study is required to validate the importance of this molecular technique in the multimodality therapy for HCC.

We showed that the detection of HCC (71.4% sensitivity and 100% specificity) is feasible by screening the serum samples with a profile of 19 microsatellite markers. However, a recent study reported that around 50% of the cirrhotic nodules are monoclonal and already have chromosomal aberrations (Yeh *et al*, 2001). Whether the aberrations are also present in the serum DNA is currently unknown. Wang *et al* (2000) reported 87% of sensitivity in detection of p15/p16 methylation in the circulating DNA of HCC patients. Although the results seem to be better than we have in monitoring the profile of microsatellite markers, both the ability of differential diagnosis of HCC from CC and their value in patients having AFP below reference range were not examined. As a result, the impact of these observations in the development of HCC and/or cell type-specific genetic markers needs to be clarified in a large cohort of liver disease patients.

In conclusion, we have determined a profile of molecular markers appropriate for differential diagnosis of primary liver cancer in the serum. The discovery may permit a high-throughput screening of hepatocellular carcinoma at an early, and potentially resectable, stage of disease.
